# Differential Response of Ileal and Colonic Microbiota in Rats with High-Fat Diet-Induced Atherosclerosis

**DOI:** 10.3390/ijms231911154

**Published:** 2022-09-22

**Authors:** Lingmiao Wen, Wei Xiong, Guihua Wei, Liudai Zhang, Yanjun Liu, Tinglan Zhang, Alvin Altamirano, Qiaozhi Yin, Tiane Zhang, Zhiyong Yan

**Affiliations:** 1 School of Life Science and Engineering, Southwest Jiaotong University, Chengdu 610031, China; 2 Institute of Chinese Materia Medica, China Academy of Chinese Medical Sciences, Beijing 100700, China; 3 Department of Chemistry and Biochemistry, Northern Arizona University, Flagstaff, AZ 86011, USA; 4 School of Basic Medicine, Chengdu University of Traditional Chinese Medicine, Chengdu 610075, China

**Keywords:** atherosclerosis, ileal microbiota, colonic microbiota, high-fat-diet, gut

## Abstract

Growing evidence suggests that gut microbiota are associated with atherosclerosis (AS). However, the functional heterogeneity of each gut segment gives rise to regional differences in gut microbiota. We established a rat model of AS by feeding the rats a high-fat diet for a long period. The pathological and microbiota changes in the ileum and colon of the rats were examined, and correlations between AS and microbiota were analyzed. The aortic mesothelium of the experimental rats was damaged. The intima showed evident calcium salt deposition, indicating that the AS rat model was successfully developed. We noted varying degrees of pathological damage in the ileum and colon of the experimental rats. The 16S rDNA high-throughput sequencing showed significant differences in α-diversity, β-diversity, and microbiota comparisons in the ileum and colon. Furthermore, the ileum and colon of AS rats showed varying degrees of intestinal microbiota disturbance. This article contributes to the study of the relationship between the microbiota in different regions of the gut and AS, and provides new approaches in gut microbiota intervention for the treatment of AS.

## 1. Introduction

Atherosclerosis (AS) is a chronic inflammatory disease with pathological features such as abnormal lipid metabolism, macrophage foaming, and plaque deposition. It involves various pathological processes, including lipid metabolism disorder, inflammatory response, platelet aggregation, proliferation, and migration of vascular smooth muscle cells, and is a major cause of blood clots, ischemic heart disease, and stroke [1,2]. Recently, there has been an increase in the prevalence of risk factors for cardiovascular and cerebrovascular diseases. AS and its complications continue to be the leading cause of morbidity and mortality worldwide, negatively impacting human life and health. Its pathology and pathogenesis are complex and are primarily attributed to abnormal lipid metabolism, inflammatory responses, and endothelial injury [3,4]. Despite the widespread use of statin lipid-lowering therapies as first-line clinical treatments, which have reduced the mortality rate associated with coronary heart disease, the global burden of the disease remains high. The gut microbiota, also known as the “second human genome”, participate in and influence the body’s metabolic processes; they are known as “multifunctional organs” and have been associated with a number of diseases [5,6]. Some recent studies have discovered an association between gut microbiota and obesity, cardiovascular disease, neurological disease, tumors, and other diseases [7,8,9,10,11,12]. Differences can be noted in the gut microbiota of individuals, which are influenced by factors such as age and environmental factors [13,14,15]. However, changes in the composition and function of microbiota result in lipid metabolism disorders and inflammatory reactions, focusing primarily on promoting the occurrence and development of AS through metabolic and nonmetabolic pathways. Improving the composition and function of gut microbiota has a significant value and emerged as a new target for the prevention and treatment of AS [16]. The mammalian gut is divided into sections called the jejunum, ileum, cecum, and colon. The distribution of microbiota in different regions of the gastrointestinal tract varies in terms of species and is influenced by regional oxygen level, nutrient bioavailability, pH value, BAs, mucus, and immune factors. The number of microbiota in the body gradually increases with the direction of the intestinal tract; the ileum and colon are estimated to contain the most microbiota, 10^8^/mL and 10^12^/mL, respectively [17].

Studies on the relationship between AS and gut microbiota are currently based on colon or fecal microbiota samples, which primarily contain colonic microorganisms, whereas small intestinal microorganisms are scarce. Although previous studies have focused on AS and gut microbiota, the microbial variation in different sections of the intestine remains unknown [18,19]. Therefore, an animal model of AS was established in this study to investigate the specific changes in the ileum and colonic microbiota in the course of atherosclerotic lesions and promote future research on the intervention or treatment of AS with gut microbiota.

## 2. Results

### 2.1. Analysis of Weight, Lipid, and Lipopolysaccharide Changes of AS Model Rats

**Figure 1:**
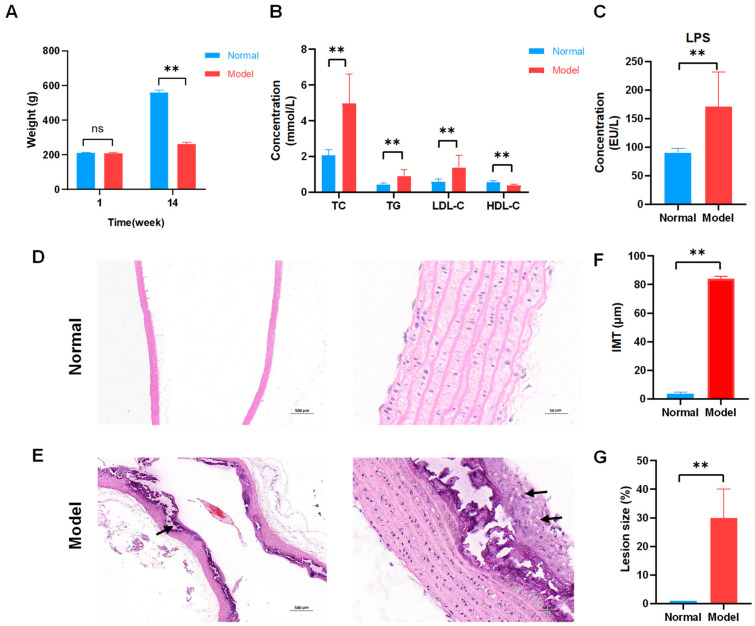
Successful establishment of atherosclerosis (AS) model in rats fed high-fat diets. (**A**) Body weight of rats during fourteen weeks (*n* = 8). (**B**) Serum lipid levels. Normal and model groups (*n* = 8). (**C**) Lipopolysaccharide (LPS) levels. Normal and model groups (*n* = 8). ** *p* < 0.01, ns *p* > 0.05. (**D**) Representative pathological changes on aortic pathology of rats in the normal group (×40, ×400). The clear structure of all layers of blood vessels and intact intima. (**E**) Representative pathological changes on aortic pathology of rats in AS model group (×40, ×400). Localized endothelial cells were detached in the vessels, and irregularly shaped smooth muscle cell nuclei were seen with foam cells (black arrow) and large calcium salt deposits (black arrow). (**F**) Aortic intima–media thickness (IMT) was measured in μm standard units, *n* = 3, ** *p* < 0.01. (**G**) Atherosclerotic lesion size (%), *n* = 3, ** *p* < 0.01.

As the experiment progressed, body weight records were examined (Figure 1A). No significant differences were found between the normal and model groups before building the AS model. After modeling, a significant difference was noted between the two groups. During modeling, modeled rats fed high-fat chow did not eat properly, a situation that could be attributed to the bitter taste of propylthiouracil, as well as to the addition of lipids, which affected the mouthfeel of the chow [20]. The results of lipid measurements showed that the serum levels of total cholesterol (TC), triglyceride (TG), and low-density lipoprotein (LDL-C) increased and that of high-density lipoprotein (HDL-C) decreased in the AS group, with highly significant differences with the normal group (*p* < 0.01; Figure 1B). LPS results showed that serum lipopolysaccharide (LPS) levels were significantly higher in the AS group compared with the normal group (*p* < 0.01; Figure 1C).

### 2.2. Analysis of Pathological Changes of AS Model Rats

Hematoxylin and eosin (HE) staining was performed to visualize atherosclerotic lesions. The aortal morphology was normal in the normal group, and smooth muscle cells were arranged neatly without pathological changes (Figure 1D). Foam cells were observed in the rat model group’s aorta tissues. Localized endothelial cell loss, endothelial fracture, uneven intima thickness, irregular shape of smooth muscle cell nuclei, and large calcium salt deposits were observed in the model group’s aorta tissues (Figure 1E). The aortic intima–media thickness (IMT) and atherosclerotic lesions size (%) were determined using image analysis software to further analyze the degree of atherosclerosis development. The IMT was larger for the model group (Figure 1F). Atherosclerotic lesions were severer in the model group (Figure 1G). These results showed that a high-fat diet successfully established a rat AS model.

### 2.3. Analysis of Histopathological Changes in the Ileum and Colon of AS Model Rats

**Figure 2:**
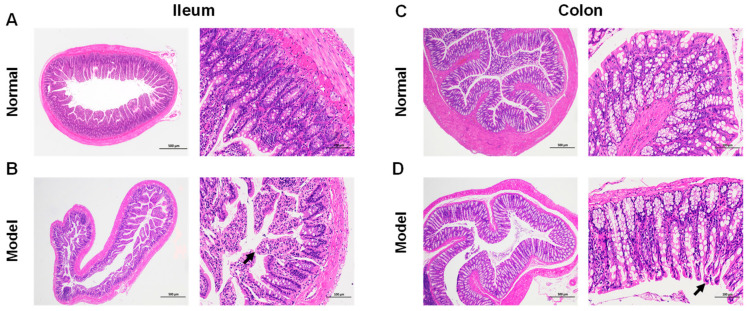
Pathological changes in the ileum and colon of AS model rats. (**A**) Representative images of hematoxylin and eosin (HE) staining of the normal group of the ileum (×40, ×200). The degree of histological lesions was graded on a five-point scale, with no or minimal lesions scored as 0; mild or minimal lesions scored as 1; moderate or intermediate lesions scored as 2; severe or multiple lesions scored as 3. The normal group in the ileum was scored as 0. (**B**) Representative images of HE staining of the model group of the ileum (×40, ×200). Mucosal epithelial cells were shed from the ileum, exposing the lamina propria of the intestinal villi (black arrow). Histopathology score as 3. (**C**) Representative images of HE staining of the normal group of the colon (×40, ×200). Histopathology score as 0. (**D**) Representative images of HE staining of the model group of the colon (×40, ×200). Showed a slight loss of mucosal epithelial cells (black arrow). Histopathology score as 1.

The histopathology of the intestine was examined using HE staining to determine the extent of damage in the ileum and colon of AS rat models. The ileum and colon tissues were clearly structured in the normal group. The mucosal epithelium and muscular layer were intact; the ileum intestinal villi were abundant and regularly arranged (Figure 2A,C). Mucosal epithelial cells were shed from the ileum of the model group (IM), exposing the lamina propria of the intestinal villi (Figure 2B). The colon of the model group (CM) showed a small amount of mucosal epithelial cells shed (Figure 2D). This suggests that pathological changes occurred in both the ileum and colon of the AS rat model, with more obvious changes in the ileum.

### 2.4. Metadata and Sequencing of the Gut Microbiota

A total of 568,652 clean tags were obtained from 16 samples (normal group: four ileum and four colon samples; model group: four ileum and four colon samples), with 550,281 effective tags screened. OTU clustering analysis was performed at 97% similarity, where the normal group contained 207 OTUs for the ileum and 380 OTUs for the colon; in the model group, there were 815 OTUs for the ileum and 196 OTUs for the colon. The values of good’s coverage of all libraries were >99%, indicating a low chance of not being measured in the sample sequence.

### 2.5. Differences in the Gut Microbiota Composition of the Ileum and Colon of Normal Rats

**Figure 3:**
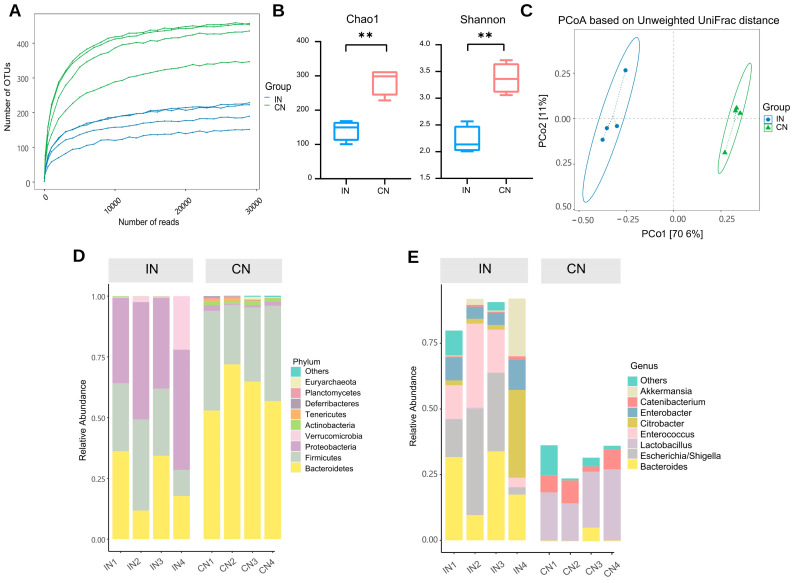
The microbiota of the ileum and colon was different in normal rats. (**A**) Rarefaction Curve. The horizontal coordinate is the number of sequencing strips randomly selected from the sample, the vertical coordinate is the number of OTUs calculated based on this number of sequencing strips, and the lines of different colors represent different samples. (**B**) Alpha diversity. ** *p* < 0.01. (**C**) Beta diversity. PCoA based on unweighted UniFrac. Dots represent samples, colors represent groups, horizontal coordinates indicate the first principal coordinate, vertical coordinates indicate the second principal coordinate, and percentages indicate the contribution of the axes to the variance of the samples. Ellipse indicates 95% confidence intervals for each group. (**D**) The distribution of the microbiota with the high abundance at the phylum level. (**E**) The distribution of the microbiota with the high abundance at the genus level. IN: the ileal microbiota of the normal group; CN: the colonic microbiota of the normal group.

The normal group’s ileum (IN) and colon (CN) microbiota were studied in the analysis of the characteristics of gut microbiota. First, a rarefaction curve was created to investigate the trend of species richness in samples with increasing sequencing depth (Figure 3A). The flattening of the curve indicates extensive sequencing depth coverage of the sample species and that increasing the data volume produces very few low-abundance species.

Then, the α-diversity was compared and expressed using the Chao1 index, which measures species richness, and the Shannon index, considering the number and evenness of species. The colon showed significantly higher Chao1 and Shannon indices than the ileum (*p* < 0.01; Figure 3B). Subsequently, to explore the diversities of the microbiomes at different locations, the β-diversity analysis was applied. Principal coordinate analysis (PCoA) was performed based on unweighted UniFrac metric. The results revealed that the ileal and colonic microbiota were distinct (Figure 3C). This showed that the microbiota of the ileum and colon of normal rats varied significantly, with the colonic microbiota having significantly more species and diversity than the ileal microbiota.

We further investigated the taxonomic distribution of abundant microbiota. *Proteobacteria* was the most abundant phylum in the ileum, accounting for 42.55% of the relative abundance. *Firmicutes* (25.99%) and *Bacteroidetes* (25.01%) were the second and third most dominant phyla in the normal group, respectively. However, the most important phyla in the colon were *Bacteroidetes* (62.78%), *Firmicutes* (34.44%), and *Proteobacteria* (1.27%; Figure 3D). The most important microbiota genera in the ileum were *Bacteroides* (23.13%), *Escherichia/Shigella* (21.83%), and *Enterococcus* (16.17%). However, the most important microbiota genera in the colon were *Lactobacillus* (20.26%) and *Bacteroides* (1.48%; Figure 3E).

### 2.6. Differences in the Gut Microbiota Composition of the Ileum and Colon of AS Model Rats

**Figure 4:**
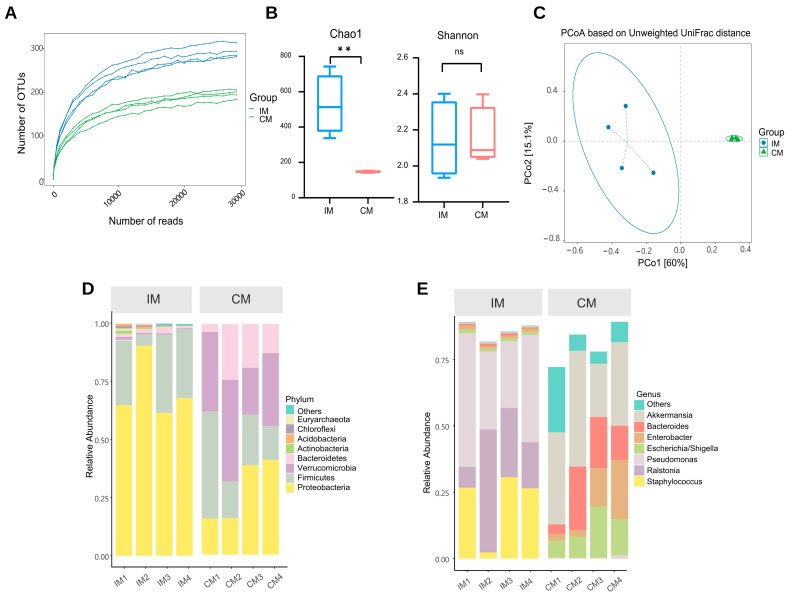
The microbiota of the ileum and colon was different in AS model rats. (**A**) Rarefaction curve. The lines of different colors represent different samples. (**B**) Alpha diversity. ** *p* < 0.01, ns *p* > 0.05. (**C**) Beta diversity. (**D**) The distribution of the microbiota with the high abundance at the phylum level. (**E**) The distribution of the microbiota with the high abundance at the genus level. IM: the ileal microbiota of the AS model group; CM: the colonic microbiota of the AS model group.

The characteristics of the AS model group’s gut microbiota were studied. Sequencing depth is sufficient (Figure 4A). In the AS model, the Chao1 index was significantly higher in the ileum than in the colon (*p* < 0.01). The ileum showed a lower Shannon index than the colon, but this was not statistically significant (Figure 4B). The β-diversity results show that the microbiota composition in the ileum samples differed significantly from that in the colon (Figure 4C). This suggests that although the overall composition of the microbiota remains significantly different, the diversity of both ileal and colonic microbiota in AS rat models is decreasing. We investigated the taxonomic distribution of the numerically abundant microbiota in two gut locations. At the phylum level in the model group, the ileum had a high relative abundance of *Proteobacteria* (72.62%), *Firmicutes* (25.11%), and *Bacteroidetes* (1.26%), whereas the colon had a high relative abundance of *Verrucomicrobia* (32.49%), *Proteobacteria* (27.60%), and *Firmicutes* (24.52%; Figure 4D). Furthermore, at the genus level in the model group, the important microbiota genera in rat colon were *Akkermansia* (32.48%). However, the important microbiota genera in the rat ileum were *Pseudomonas* (36.31%; Figure 4E).

### 2.7. Differences in the Ileal Microbiota Composition between Normal and AS Model Rats

**Figure 5:**
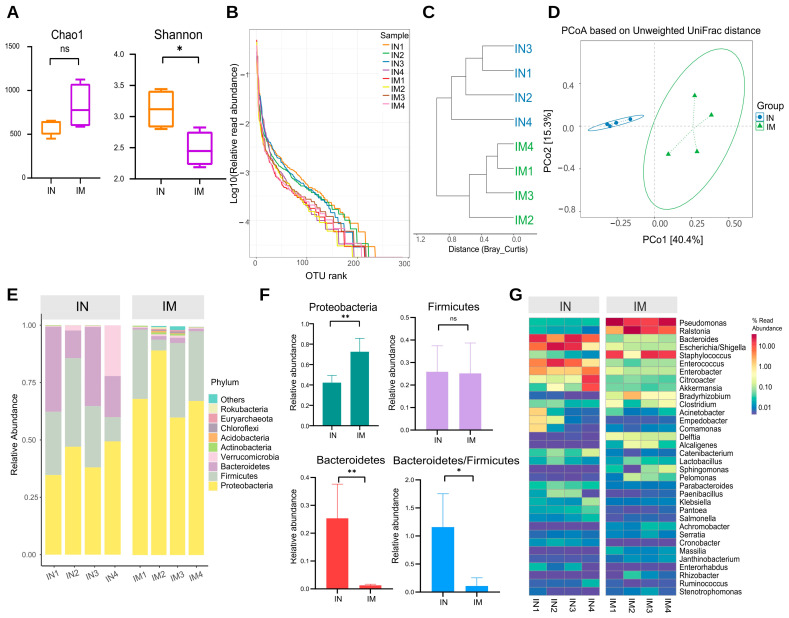
The microbiota diversity in the normal and AS model groups of the ileum was different. (**A**) Alpha diversity. * *p* < 0.05, ns *p* > 0.05. (**B**) Rank–abundance curve. (**C**) Cluster tree based on Bray–Curtis distances. (**D**) PCoA based on unweighted UniFrac. IN: the ileal microbiota of the normal group; IM: the ileal microbiota of the AS model group. (**E**) The distribution of the microbiota with the high abundance at the phylum level. (**F**) The distribution of the dominant microbiota with the highest abundance at the phylum level. ** *p* < 0.01, * *p* < 0.05, ns *p* > 0.05. (**G**) Heatmap of high abundance at the genus level. IN: the ileal microbiota of the normal group; IM: the ileal microbiota of the AS model group.

Changes in ileal microbiota profile in AS were analyzed along with microbiota of the ileum in the normal (IN) and model (IM) groups. The α-diversity results showed that the Chao1 index was increased in the IM group compared with the IN group, but the difference did not show a significant change (Figure 5A). The Shannon indices were significantly lower in the IM group than in the IN group (*p* < 0.05). To investigate trends in species abundance and homogeneity, a rank–abundance curve was used (Figure 5B). The curve’s width reflected species abundance in the horizontal direction. The IM group had a smaller horizontal range than the IN group, indicating lower abundance. The smoothness of the curve reflects species homogeneity, with a steeper trend in the IM group indicating a more heterogeneous distribution of species.

Cluster analysis and graphing were performed to visually compare the similarity among different samples (Figure 5C). The findings revealed that samples from the same group clustered in the same branch, indicating that the parallel sample communities within the group were similar in structure and homogeneity. The IN and IM groups had a distinct phylogenetic composition. Variation in the microbial community composition and similarity between the samples was then investigated at the OTU level, and the β-diversity of each group was calculated (Figure 5D). The ileal microbiota composition differed between the normal and model groups. The microbiota of the IN and IM groups formed two distinct clusters according to PCoA of weighted UniFrac distance.

The phylum and genus of the microorganisms were analyzed to further investigate the characteristics of the ileal microbiota. *Proteobacteria*, *Firmicutes*, *Bacteroidetes*, *Verrucomicrobia*, and *Actinobacteria* represented the top five phyla in terms of relative abundance. *Proteobacteria* were the most abundant in the IN and IM groups, with an absolute predominance, at 42.3% and 72.6% relative abundance, respectively, followed by *Firmicutes*, with 25.9% and 25.2% abundance. *Bacteroidetes* accounted for 25.3% of the ileal gut microbiota in the IN group but only 1.3% in the IM group (Figure 5E). In the ileum samples, *Proteobacteria* were significantly increased in the model group compared with the normal group (*p* < 0.01), *Bacteroidetes* were significantly decreased (*p* < 0.01), and the ratio of *Bacteroidetes/Firmicutes* was decreased (*p* < 0.05; Figure 5F). At the genus level, *Pseudomonas*, *Ralstonia*, *Bacteroides*, *Escherichia/Shigella*, and *Staphylococcus* were the top five groups in terms of relative abundance. The abundance of *Pseudomonas* and *Ralstonia* was significantly higher in the IM group than in the IN group (*p* < 0.01 and *p* < 0.05), whereas the abundance of *Bacteroides* and *Escherichia/Shigella* was significantly lower in the IM group than in the IN group (Figure 5G).

**Figure 6:**
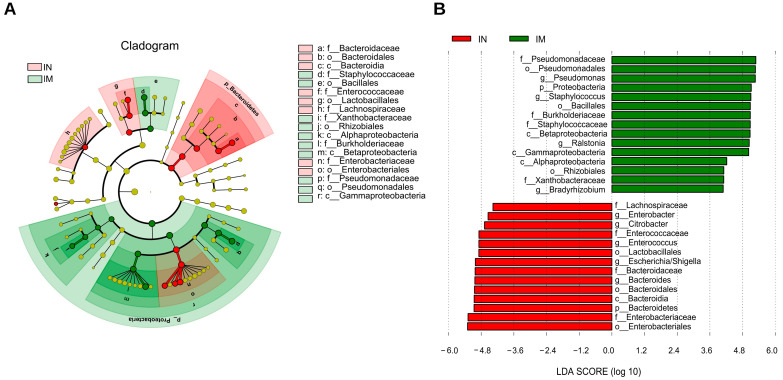
The microbiota comparisons in the normal and AS model groups of the ileum was different. (**A**) Cladogram of Lefse. It is used to show the evolutionary laws of species branching that play an important role in each group of samples. (**B**) LDA score of Lefse. The horizontal coordinate represents the size of the LDA score; the longer the length of the bar, the more important this group is. The colors represent different groups, indicating that within this group these taxa have significantly enriched. IN: the ileal microbiota of the normal group; IM: the ileal microbiota of the AS model group.

A linear discriminant analysis effect size (LEfSe) analysis was used to identify bacterial genera that were differentially enriched to identify the important gut microbiota that varied significantly between groups. By screening the results for linear discriminant analysis scores of >3. In the IM group, the significantly different species included *Pseudomonadacease*, *Proteobacteria*, *Burkholderiaceae*, *Staphylococcaceae*, *Gammaproteobacteria*, and others (Figure 6A,B).

### 2.8. Differences in the Colonic Microbiota Composition between Normal and AS Model Rats

**Figure 7:**
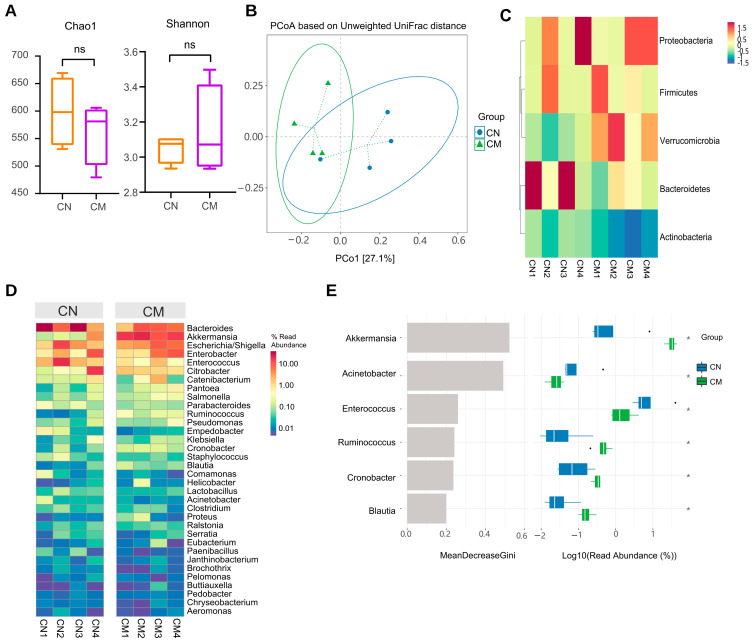
The microbiota in the normal and AS model groups of the colon was different. (**A**) Alpha diversity. ns *p* > 0.05. (**B**) The beta diversity. (**C**) The distribution of the microbiota with the high abundance at the phylum level. (**D**) Heatmap of high abundance at the genus level. (**E**) Random forest analysis. The horizontal coordinates of the left panel are the mean reduction in the Gini index, the vertical coordinates are the taxonomic information of the genus, and the right panel is a box plot of the abundance of taxa in different groups, where the * signs on the right represent the degree of significance of the differences. * *p* < 0.05. CN: the colonic microbiota of the normal group. CM: the colonic microbiota of the AS model group.

To evaluate the effect of AS on the phylogenetic composition of microbial communities in different parts of the gut, the microbiota of the CN and the CM groups were further compared. The colon of the normal and model groups did not differ in terms of α-diversity (*p* > 0.05; Figure 7A). PCoA revealed local clustering between the colon’s normal and model groups, but there was general overlap (Figure 7B).

The most prevalent phyla in the CN and CM groups, according to the results of the OTU annotation analysis, were *Bacteroidetes*, *Proteobacteria*, *Firmicutes*, and *Verrucomicrobia*; the most predominant genera were *Bacteroides*, *Akkermansia*, *Escherichia/Shigella*, *Enterobacter*, *Enterococcus*, and *Citrobacter* (Figure 7C,D). We also used machine learning analysis to identify microbiota genera that were differentially enriched between the two communities (Figure 7E). Along with variance tests, random forest analysis can be used in machine learning analysis. Random forest is a popular classification method in machine learning. It is a classifier with multiple decision trees that can handle large numbers of input variables, assess their importance, and balance data inaccuracies. Hence, it is used to assess the taxonomic importance of species between groups to facilitate comprehensive analyses in conjunction with the species’ sequence abundance as well as physiological and biochemical properties. The mean decreases in the Gini index for *Akkermansia* were rapid, indicating greater taxonomic importance between groups.

### 2.9. Correlation Analysis between Gut Microbiota and Physicochemical Factors

**Figure 8:**
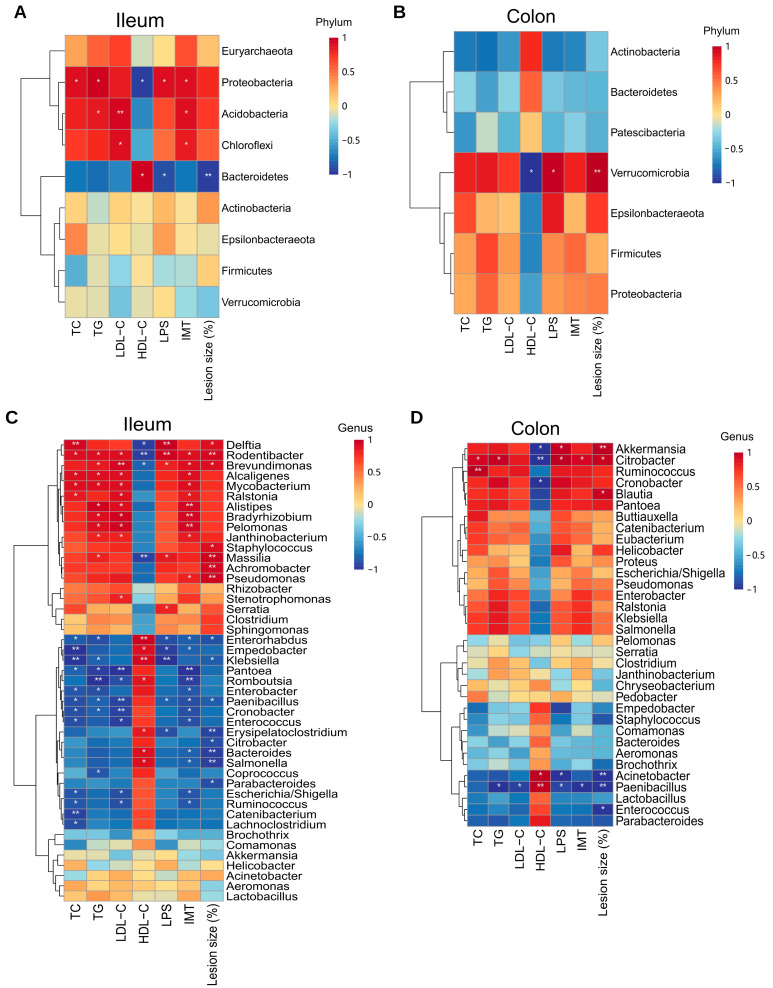
Correlation analysis between ileal and colonic microbiota and AS development. (**A**) Correlation analysis of ileal microbiota with AS at the phylum level. (**B**) Correlation analysis of ileal microbiota with AS at the genus level. (**C**) Correlation analysis of colonic microbiota with AS at the phylum level. (**D**) Correlation analysis of colonic microbiota with AS at the genus level. Red represents positive correlation and blue represents negative correlation. ** *p* < 0.01, * *p* < 0.05.

Using Spearman’s rank correlation coefficient, the correlation between gut microbiota and AS in two different intestinal sections, ileal and colonic, was analyzed (Figure 8). At the phylum classification level, ileal gut microbiota was significantly correlated with four phyla, with *Proteobacteria* being significantly positively correlated with TC, TG, IMT, and LPS, and negatively correlated with HDL-C. *Bacteroidetes* were significantly and negatively correlated with LPS and atherosclerotic lesion size (%). Colonic gut microbiota was shown to be significantly correlated with *Verrucomicrobia* only, negatively correlated with HDL-C, and positively correlated with LPS and atherosclerotic lesion size (%). At the genus classification level, ileal gut microbiota was significantly associated with thirty-five genera and colonic gut microbiota were significantly associated with eight genera. In the ileum, *Escherichia/Shigella*, *Paenibacillus*, *Cronobacter*, etc., had a correlation with lipid levels; *Rodentibacter*, *Pseudomonas*, *Brevundimonas*, etc., had a positive correlation with pathological changes; *Brevundimonas*, *Rodentibacter*, *Serratia,* etc., had a correlation with LPS. In the colon, *Citrobacter*, *Paenibacillus*, etc., correlated with the development of AS.

## 3. Discussion

In this study, we showed that the serum levels of TC, TG, and LDL-C were higher and HDL-C was lower in the atheromatous model rats. Studies have shown that lipid abnormalities are a major risk factor in the development of atherosclerosis. The higher the serum levels of TC, TG, and LDL and the lower the HDL levels, the greater the risk of developing AS [21]. We discovered various pathological changes in rat ileal and colonic tissues due to high-fat diet-induced AS. The ileal mucosal layer had more severe mucosal epithelial damage than the colon. Consistent with our findings, a previous study showed that feeding a high-fat diet for a prolonged period leads to a local inflammatory response in the intestine, resulting in damage to the intestinal barrier and increased permeability [22]. Previous studies may explain how a high-fat diet increases plasma lipopolysaccharide (LPS) levels by altering the gut microbiota composition, inducing intestinal barrier dysfunction, and increasing gut permeability [23]. LPS embedded in gut enterocyte-derived chylomicrons crosses the intestinal barrier and enters the bloodstream, where it binds to Toll-like receptors [24]. LPS triggers the proinflammatory response of the innate immune system, resulting in endothelial dysfunction, atherosclerotic plaque formation, and rupture [25]. Increasing evidence indicates that gut microbiota can either directly or indirectly contribute to the development and progression of AS [26]. Thus, our discussion focuses on gut microbiota and attempts to differentiate the ileal and colonic microbiota of the normal and model groups, and to analyze the correlation between the microbiota and the development of AS.

Separate studies were conducted on the various anatomical sites of the intestine in the same group (IN vs. CN and IM vs. CM). In the normal group (IN vs. CN), α-diversity indicated a higher abundance and diversity of the colon community. Likewise, β-diversity indicators were used to visualize the differences, with a clear distinction between ileal and colonic microbiota composition of rats in the normal groups. Previous research has shown that microbiota abundance gradually increases from the ileum to colon in the same body [27]. One possible explanation for the low microorganism count of the ileum compared with that of the colon is its purposeful design for macro- and micronutrient absorption and immune regulation [28]. The pH, oxygen levels, and immune effectors are some of the several factors that influence the formation of the gut microbiota structure [29]. The ileal microbiota appears to have a higher abundance of *Proteobacteria*, *Enterococcus*, and *Escherichia/Shigella*, whereas the abundance of *Bacteroidetes* and *Lactobacillus* was in higher in the colonic microbiota. This result was consistent with that of a previous study [30]. The colonic and ileal microbiota also differed among the rats in the model group (IM vs. CM). The development of AS may be associated with a disruption in microbiota homeostasis, and the relative abundance of *Desulfovibrio spp.* was higher in the colon than in the ileum. Zhang et al. showed that the abundance of endotoxin-producing Desulfovibrio damages the gut barrier function and results in high levels of circulating LPS [31].

Our study reveals differences in microbial composition of the ileum and colon between the normal (IN vs. CN) and model (IM vs. CM) groups. These findings suggest that the gut microbiota composition in AS rat models was altered; this alteration is noted in both the ileum and colon. Therefore, we sought to extend our analysis in the microbiome of the normal and model groups in the same intestinal segment to investigate what specifically is altered in the gut microbiota of the ileum and colon in the AS model (IN vs. IM; CN vs. CM) and which microbiota are correlated with the progression of AS.

First, we analyzed the gut microbiota of ileum samples (IN vs. IM). The total number and abundance of ileal microbiota in the IM group varied significantly compared with the IN group. The phenomenon of high species abundance but uneven distribution and significantly reduced diversity and homogeneity in the ileal gut microbiota of AS rat models suggested a disturbance in the ileal microbiota. The stability of the gut microbiota contributes to the intestinal mucosal barrier and nutrient uptake function. It maintains and improves the immune system by regulating metabolism and preventing pathogenic microorganisms and their metabolites from entering the circulatory system [32].

At the phylum level, the relative abundance of *Proteobacteria* was significantly higher in the model group than in the normal group (*p* < 0.05), and we found a significant positive correlation between *Proteobacteria* and blood lipid levels. *Proteobacteria* are mostly Gram-negative bacteria that produce a considerable amount of LPS in the intestine. Caesar et al. reported that high levels of intestinal LPS lead to a local inflammatory response in the intestine. It disrupts the intestine’s barrier function by widening the gaps between intestinal epithelial cells, followed by the entry of LPS and microorganisms into the body’s circulation through the damaged intestinal epithelium, thus triggering or exacerbating the AS lesions [33]. Moreover, the relative abundance of *Bacteroidetes* in the ileum of AS rat models were significantly reduced (*p* < 0.05) in our study, which is consistent with the finding of Karlsson’s study [34]. In addition, we found a significant negative correlation between *Bacteroidetes* and atherosclerotic lesion (%) by correlation analysis. Evidence suggests that short chain fatty acids are produced by *Bacteroidetes* and act on free fatty acid receptors to prevent invasion by pathogenic microorganisms and improve the stability of AS plaques by reducing the expression of the chemotactic protein, adhesion molecule, and matrix metalloproteinase at AS plaque sites, thus inhibiting macrophage migration and collagen deposition [35]. AS rat models showed a significant (*p* < 0.05) decrease in the *Bacteroidetes* to *Firmicutes* ratio. Evidence indicates that high-fat diets disrupt glucolipid metabolism, causing an imbalance in gut microbiota composition and resulting in a lower *Bacteroidetes* to *Firmicutes* ratio, which is associated with an increase in the IMT [36]. The LEfSe results revealed that the beneficial bacteria *Enterococcus* were significantly enriched in the normal group. Previous research found that in ApoE^−/−^ genic rats fed with a high-cholesterol diet, *Enterococcus* reduces the size of AS plaques as the *Enterococcus* population is negatively associated with the levels of TC, non-HDL cholesterol, and autoantibodies against oxidized LDL as well as the size of the lesions [37].

We investigated the colonic microbiota (CN vs. CM) to further evaluate the changes in the microbiota of different anatomical intestinal regions of the AS model. Surprisingly, there were no significant differences in the α- and β-diversities of the colonic microbiota between the normal and model groups. The model group differed from the normal group; the differences were not significant for *Bacteroidetes*, *Proteobacteria*, and *Firmicutes* but were significant for *Verrucomicrobia*. Our findings were supported by the results of machine learning analysis, which revealed the enrichment of *Akkermansia* in the colon of the model group. According to one study, the relative abundance of *Akkermansia* in the atherosclerotic colonic microbiota was significantly higher in rats fed with a high-fat diet than in normal rats. On the other, we found that *Citrobacter* was positively correlated with IMT, which is consistent with the previous findings [38]. Our findings also show that *Lachnospiraceae* was enriched in the colon of the model group. Previous studies demonstrated that trimethylamine N-oxide produced by *Lachnospiraceae* induces vascular inflammation by activating inflammatory pathways and promoting leukocyte adhesion to endothelial cells, leading to AS [39].

Finally, the analysis of the correlation between microbiota and the development of AS showed that more microbiota in the ileal gut correlated with lipid levels (TC, TG, LDL-C, HDL-C) associated with the development of AS, as well as with IMT and atherosclerotic lesion size (%), which reflect the degree of pathological changes in AS, but only a few microbiotas in the colonic gut correlated with each index.

## 4. Materials and Methods

### 4.1. Animals

A total of 16 specific pathogen-free, 7-week-old Sprague–Dawley male rats weighing between 170–190 g were used in the study. Sichuan Chengdu Dashuo Experimental Animal Co., Ltd., China provided the experimental animals, and the production license is SCXK (Chengdu, CHN) 2020-030. The animals were raised in the Animal Laboratory of Southwest Jiaotong University’s School of Life Science and Engineering. The breeding temperature was 22 °C ± 2 °C, the relative humidity was 50% ± 10%, and the light–dark cycle was 12 h. For a week of acclimation, the rats were fed adaptively and given unlimited access to food and water. Ketamine (75 mg/kg) and xylazine (5 mg/kg) were administered intraperitoneally for anesthesia. Rats were euthanized by intravenous injection of a lethal dose of pentobarbital sodium (100 mg/kg). Animal care and study protocols were approved by the institutional animal ethics committee at Southwest Jiaotong University (approval no. SWJTU-2010-001) with the approval date of November 10, 2020.

### 4.2. Establishment of Atherosclerotic Animal Model

After 7 days of adaptive feeding, the rats were randomly divided into normal (*n* = 8) and model groups (*n* = 8). In this experiment, the AS model was established using a high-fat diet. During the modeling period, the model group was fed with a high-fat diet (3.5% cholesterol +0.5% sodium cholate +0.2% propylthiouracil +5% sugar +10% lard +80.8% basal diet) [40], and the normal group was fed with a normal diet (58% carbohydrate +24% protein +18% fat; Sichuan Chengdu Dashuo Experimental Animal Co., Ltd.,Chengdu, CHN; 20200501) for 14 weeks. The body weights of the rats were recorded at weeks 1 and 14.

### 4.3. Enzyme Linked Immunosorbent Assay

Blood was collected from the femoral artery in centrifuge tubes. After 2 h at room temperature, the blood was centrifuged at 2 °C – 8 °C for 15 min at 3000 rpm. The supernatant was removed and frozen at −80 °C. Enzyme-linked immunosorbent assay (ELISA) kits were used for the determination of total cholesterol (TC; Nanjing Jiancheng Biotechnology Co.,Ltd., Nanjing, CHN; A111-1-1), triglyceride (TG; Nanjing Jiancheng Biotechnology Co.,Ltd., Nanjing, CHN; A110-1-1), high-density lipoprotein (HDL-C; Nanjing Jiancheng Biotechnology Co.,Ltd., Nanjing, CHN; A112-1-1), low-density lipoprotein (LDL-C; Nanjing Jiancheng Biotechnology Co.,Ltd., Nanjing, CHN; A113-1-1), and lipopolysaccharide (LPS; Nanjing Jiancheng Biotechnology Co.,Ltd., Nanjing, CHN; SU-B31044) in serum. The procedure followed was as follows: the sample was added, followed by enzyme conjugates; then, the chromogenic substrate was added, and the reaction was stopped. The optical density was measured at 510 nm on an enzyme labeling instrument (Perlong; Beijing, CHN; DNM9602G) [41].

### 4.4. Analysis of Atherosclerotic Lesions

The rats were sacrificed, and the aorta, ileum, and colon were rapidly separated and placed on an ice plate. The contents of the ileum and colon were washed. Then, the tissue was fixed in 10% paraformaldehyde, dehydrated, paraffin wax-embedded, and sliced. The slices were dewaxed with xylene (Servicebio; Wuhan, China; SCR10023418), 100% ethanol, and 75% ethanol (Servicebio; Wuhan, China; SCR100092683). Then, sections were stained with hematoxylin and eosin (HE; Servicebio; Wuhan, China; G1004), dehydrated and transparent with 100% ethanol, treated with xylene, and sealed with neutral gum (Servicebio; Wuhan, China; SCR10004160). Finally, the slices were observed under an upright optical microscope at different magnifications (×40, ×400; Nikon; Tokyo, Japan; Nikon Eclipse E100) and imaged with an image system (Nikon; Tokyo, Japan; Nikon DS-U3) [42,43]. The aortic intima–media thickness (IMT) and atherosclerotic lesion size (%) were measured using Image-Pro Plus 6.0 software (Media Cybemetics; Rockville, MD, USA) on ×40 histological maps.

### 4.5. DNA Extraction, PCR Amplification, and Sequencing

Ileum and colon contents were collected in cryopreservation tubes and stored at −80 °C. Sample gDNA was purified using the Zymo Research BIOMICS DNA Microprep Kit (Zymo research; California, USA; D4301). The gDNA integrity was assessed via 0.8% agarose electrophoresis, followed by nucleic acid concentration analysis and dilution using Tecan F200. Specific primers with index sequences were synthesized. The 16S rDNA V4 region of the sample was amplified according to the sequenced region with the following amplified primer sequences: Primer5′-3′: 515F (5′-GTGYCAGCMGCCGCGGTAA-3′) and 806R (5′-GGACTACHVGGGTWTCTAAT-3′). In which, “Y”, means “C/T”; “M”, means “A/C”; “H”, means “A/C/T (not G)”; “V”, means “A/C/G (not T)”.

Polymerase chain reaction (PCR) was then performed using diluted genomic DNA as the template and specific primers with a barcode, depending on the sequencing region. Each 25 μL reaction comprised 1X PCR buffer, 1.5 mM MgCl2, 0.4 μM dNTPs, 1.0 μM each of forward and reverse primers, 0.5 U TOYOBO KOD-Plus-Neo enzyme (Toyobo; Osaka, Japan; KOD-401B), and 10 ng of the template. The PCR program was set as follows: initial denaturation at 94 °C for 1 min; 30 cycles of denaturation at 94 °C for 20 s, annealing at 54 °C for 30 s, and extension at 72 °C for 30 s; and a final elongation step at 72 °C for 5 min [44].

Three replicates were used for each PCR sample. Afterward, the PCR products were tested, purified, and quantified. The PCR products were thoroughly mixed with a six-fold volume of loading buffer before electrophoresis on a 2% agarose gel to detect the target fragments [45]. Qualified samples were screened, and the target bands were recovered using the Zymoclean Gel Recovery Kit (Zymo Research; Irvine, CA, USA; D4008) before quantifying them using the Qubit@ 2.0 Fluorometer (Thermo Fisher Scientific; Waltham, MA, USA). Finally, they were mixed in equimolar amounts. The NEBNext Ultra II DNA Library Prep Kit for Illumina (NEB; San Diego, CA, USA; E7645L) was used to create libraries, and high-throughput sequencing was performed using the HiSeq Rapid SBS Kit v2 (Illumina; San Diego, CA, USA; 20028402) using the PE250 sequencing method.

### 4.6. Bioinformatics and Statistical Analysis

FLASH was used to splice double-ended sequences [46]. Based on the barcode, the sequence of each sample was separated from the reads [47]. Barcode sequences were truncated to obtain raw data and then controlled for quality using the QIIME (v. 1.9.0) software package [48]. Clustering, species annotation, and the construction of an evolutionary tree operational taxonomic unit (OTU) clustering were performed using the UPARSE algorithm at a 97% similarity level using the Research (http://drive.com/uparse/, accessed on 10 January 2022) software. The sequence with the highest frequency was selected as the representative sequence for each OTU and annotated for analysis based on the SILVA database [49]. PyNAST was used to compare and filter the representative sequences, and FastTree was used to build an evolutionary tree [50]. Individual samples were homogenized, and the criterion for resampling was the sample with the least amount of data. Correlation analysis between the microbiota composition and AS development was performed with sparcc analysis [51]. Statistical analysis and graphing were performed using the programming languages R (v. 3.6.0), python (v. 3.7.4), and java (v. 8).

### 4.7. Data Analysis

The experimental data were analyzed using IBM SPSS Statistics 22 and GraphPad Prism 8.0.2, and the parameters were compared between the groups using independent samples. Student’s *t*-test was used to compare the two groups. The data were expressed as means ± standard error of means ($\bar{x}$ ± SEM); the statistical significance was set at *p* < 0.05.

## 5. Conclusions

In conclusion, there were differences in ileal and colonic microbiota in the normal group that persisted with the pathological changes associated with AS. Specific changes in ileal and colonic microbiota were evaluated in depth. We found that the ileal microbes of AS rats differed significantly from those of the normal rats; however, the differences in the colonic microbiota were comparatively minor. We demonstrated that disturbances in the ileal microbiota are more closely associated with AS development than those in the colonic microbiota. Our findings paint a clear picture of the changes that occur in the microbiota at different intestinal anatomical sites and provide insights into the characteristics of microbial distribution and composition in AS rat models. Given the severity of AS, further studies are warranted to determine the effect of interventions on the ileal and colonic microbiota to develop newer and more specific therapeutic targets.

## Data Availability

The datasets generated for this study can be found in the NCBI SRA data with accession number of PRJNA804316.
